# How do children adapt their fairness norm? Evidence from computational modeling

**DOI:** 10.1371/journal.pone.0277508

**Published:** 2022-11-16

**Authors:** Frédérick Morasse, Miriam H. Beauchamp, Élise Désilets, Sébastien Hétu

**Affiliations:** 1 Department of Psychology, Université de Montréal, Montreal, Quebec, Canada; 2 Sainte-Justine Hospital Research Centre, Montreal, Quebec, Canada; 3 Department of Psychology, Université du Québec à Trois-Rivières, Trois-Rivières, Quebec, Canada; Mississippi State University, UNITED STATES

## Abstract

Adequate social functioning during childhood requires context-appropriate social decision-making. To make such decisions, children rely on their social norms, conceptualized as cognitive models of shared expectations. Since social norms are dynamic, children must adapt their models of shared expectations and modify their behavior in line with their social environment. This study aimed to investigate children’s abilities to use social information to adapt their fairness norm and to identify the computational mechanism governing this process. Thirty children (7–11 years, *M* = 7.9 *SD* = 0.85, 11 girls) played the role of Responder in a modified version of the Ultimatum Game–a two-player game based on the fairness norm–in which they had to choose to accept or reject offers from different Proposers. Norm adaptation was assessed by comparing rejection rates before and after a conditioning block in which children received several low offers. Computational models were compared to test which best explains children’s behavior during the game. Mean rejection rate decreased significantly after receiving several low offers suggesting that children have the ability to dynamically update their fairness norm and adapt to changing social environments. Model-based analyses suggest that this process involves the computation of norm-prediction errors. This is the first study on norm adaptation capacities in school-aged children that uses a computational approach. Children use implicit social information to adapt their fairness norm to changing environments and this process appears to be supported by a computational mechanism in which norm-prediction errors are used to update norms.

## Introduction

Childhood is characterized by a broad range of contexts within which social expectations often differ (e.g., home, school, sports practice, church, friend’s house). To function adequately, children must make context-appropriate social decisions in order to behave in a way that best meets the social expectations of the group [1, 2]. This social decision-making process can be facilitated by relying on social norms [3], defined as shared expectations among members of a group about the behaviors, attitudes and beliefs expected in a given situation [3, 4]. Children who exhibit difficulties in perceiving and adapting their behavior to their groups’ social norms may experience negative social consequences such as peer disapproval, negative emotions (e.g., shame, guilt), and social exclusion [4–8]. The ability to conform, enforce, but also to change one’s social norms is therefore essential for adequate social functioning during childhood [6].

From a cognitive perspective, social norms can be conceptualized as models of shared expectations based on the brain’s capacity to: 1) create a shared cognitive representation of what is expected (i.e., a social norm); 2) detect norm violations; and 3) choose the best actions to correct these violations [3, 9]. Once the cognitive representation of the norm exists, children should be able to adequately process it and therefore judge the behaviors of other group members according to the established norm, as well as choose the appropriate response when others comply with or violate this norm. If a child evaluates another group member’s behavior as non-conforming, his or her brain will detect a norm violation [9]. Work in adults has shown that these violations can be represented in the brain as a norm-prediction error signal resulting from a discrepancy between what was expected and what actually happened [9–11]. When a norm violation is detected, children can attempt to reduce the norm-prediction error in two different ways. They can try to modify their social environment through norm enforcement behaviors such as punishing the norm violator, or, they can try to modify their cognitive representation of the norm (i.e., change their social norm) in an effort to adapt to the current social environment [9]. Thus, social norm processing depends on two distinct cognitive mechanisms: detection of norm violations and norm adaptation. However, knowledge about these mechanisms in children remains limited.

Economic tasks that rely on resources distribution can be used to study social norm processing by measuring detection and response to norm violations. Several such tasks are based on the principle of fairness, a fundamental aspect of appropriate social functioning during childhood [12]. For instance, the Ultimatum Game (UG) is frequently used to evaluate social decision-making related to the fairness norm [13–16]. In the UG, a player (Proposer) has to divide a money endowment (or any other resources) with a second player (Responder). If the Responder accepts the offer, each player receives the proposed amount, however, if he rejects it, both parties receive nothing. In this task, social norms are represented by the value of the offers Responders think they should receive (i.e., their expectation) based on their own fairness norm. The UG creates a social context in which participants, playing as Responders, have to decide between self-interest (i.e., accept any sum of money) and norm enforcement (i.e., punish norm violation by rejecting the Proposer’s offer) [9] and is thus ideal to study the processing of the fairness norm.

Previous studies on fairness have found that children develop a basic understanding of what is considered a fair and an unfair division of resources around two years of age [17, 18]. This understanding continues to evolve throughout childhood, leading to changes in children’s behaviors as they age [13, 19]. For example, in a situation where they have to divide resources with another person, children aged 3 to 6 years tend to make self-centered decisions that benefit themselves at the expense of a fair division between them and their partner, whereas children aged 8 to 12 years tend to make fair offers to their partner [19]. Similar results are found regarding norm enforcement behaviors, where motivation to enforce fairness by punishing norm violations progresses from being mostly based on personal gains around 3–4 years [13–15] to being more centered around the fairness norm (regardless of potential personal gains) at 8 years [13, 14, 20]. This tendency to make social decisions that are less egocentric and more fairness-based continues to develop until at least 12 years [13–15]. Furthermore, studies using third-party punishment (i.e., punisher is not directly affected by the transgression) show comparable developmental results when punishments were administered by a third party, as well as when children were the unaffected third parties [14, 21, 22]. Together, these studies show that the ability to detect fairness violation and to enforce this social norm progressively develops and complexifies throughout childhood, where a more integrated and stable understanding of fairness seems to appear between the ages of 8 and 12, which makes this age range particularly interesting to study early social norm processing.

To be socially competent, children must be able to adapt their social norms (i.e., expectations) and behaviors to changing social environments [23, 24]. To date, few studies have specifically investigated social norm adaptation abilities in children. However, studies exploring conformity in 3- and 4-year-olds suggest that in situations where the "right answer" or "right way to act" is made explicit by adults, children are more likely to modify their behavior to conform to social expectations [25–28]. Other studies with older children have attempted to investigate behavioral adaptation using resource allocation tasks [27, 29]. In these studies, explicit social information is given to participants to investigate its influence on social decision-making. For instance, in a study by House and Tomasello [27], experimenters commented on how they think the child should divide resources (tokens) with their partner (i.e., "the right thing to do is…" or "I think you should give…"). From 6 years of age, children tend to base their allocation decisions on what the experimenter suggests. This tendency becomes more evident around the age of 7.5 years and continues to strengthen until 11 years [27]. Results from a similar study [29] showed that participants aged 9 to 18 years tend to adjust their allocation offers after obtaining information revealing the type of allocation other players of similar age make (e.g., the child will give less to another person if they see that their peers are making these types of offers).

These studies illustrate that when faced with explicit information from a third party about normative behavioral expectations, children are able to adjust their behavior in accordance with these expectations. However, some methodological limitations preclude specific conclusions regarding children’s ability to adapt their social norm when it differs from the group’s norm. In everyday life, social norms most often represent implicit expectations amongst different members of the same group [3] and children must be able to infer group norms from implicit cues in order to adjust their expectations to that norm [30]. Thus, in order to better understand the mechanism of norm adaptation in children, it is crucial that they be exposed to implicit information about the group’s norm, yet most tasks only expose children to explicit information. Furthermore, in order to study how children dynamically use this implicit social information to adapt their norm, it is necessary to expose them to a changing social environment [3, 30]. Measuring these implicit components would allow for a better representation of the complexity of real social interactions and therefore provide a more specific and ecological measure of children’s norm adaptation abilities.

A version of the UG developed and used to study social norm adaptation processes in adults seems to address some of the limitations mentioned above [9–11, 31, 32]. In this version, participants are assigned to the Responder’s role and have to accept or reject offers from several different Proposers. Throughout the task, participants are exposed to social environments characterized by different types of offers, for example relatively fair offers (e.g., around 8$ for a 20$ endowment) versus low offers (e.g., around 4$ for a 20$ endowment). Conditioning manipulations can be used to measure changes in rejection rates for fair offers after participants are presented with a series of low offers (i.e., the conditioning manipulation). Findings using this paradigm in adults indicate that average rejection rates for fair offers are influenced by such conditioning [10, 11]. This suggests that adults use the implicit information present in the social environment to adapt their internal expectations (i.e., social norms) to the context and that this adaptation can be objectified by the change in their decisions regarding the offers they receive. Importantly, in addition to providing a more specific measure of norm adaptation, this version of the UG is also tailored for model-based analysis, which has been successful in understanding underlying computational models used in various types of social decision-making [33, 34] including fairness norm adaptation [9–11, 31, 32, 35, 36]. However, these approaches have seldom been used to study social norm processing in children.

The objective of this study was to use the UG paradigm with school-age children (7–11 years; considering that they are in the time period when the understanding of fairness becomes more stable and integrated) to investigate early fairness norm adaptation. Specifically, we aimed to test children’s abilities to dynamically adapt their social norms based on implicit social information in a changing social environment and identify the computational mechanism controlling this process. We hypothesized that children’s rejection rate for fair offers would decrease after observing several low offers—indicating that they adapted their fairness norm—and that this process would be best explained by a dynamic learning computational model.

## Methods

### Participants

The current sample includes 30 typically developing children tested between the ages of 7 and 11 years (*M* = 7.90, *SD* = .85; 11 girls). Children were recruited via information pamphlets distributed to parents in local centers. Exclusion criteria were: (i) clinical diagnosis of any significant congenital, neurological, developmental, metabolic or psychiatric condition; (ii) less than 36 weeks of gestation; (iii) history of traumatic brain injury; and (iv) child and parent not fluent in French or English. This study was approved by the local research ethics committee of the Centre Hospitalier Universitaire Sainte-Justine (#2012–346) and was conducted in accordance with the declaration of Helsinki. Parents provided informed written consent prior to participation and were compensated $30 for their child’s participation.

### Measures

#### Task description

The Ultimatum Game (UG) is a two player (Proposer and Responder) game designed to evaluate social decision making in the context of social norms [37]. In its most common form, a Proposer receives an initial endowment (e.g., 20$) that he must split with a Responder, who in turn has to accept or reject the proposed split. If the Responder accepts the offer, each player receives the proposed amount, however, if he rejects it, both parties receive 0$. In this task, social norms are represented by the value of the offers Responders think they should receive (i.e., the amount they expect to receive) based on their own norms of fairness [10, 11, 31, 38]. Rejections are considered as norm enforcement signals where the Responder punishes the Proposer (at a cost for himself) for transgressing the social norm of fairness [39] and is thus often considered as altruistic punishment. In the present study, children completed a modified, computer version of the UG [10, 11, 32]. In this version, tokens were used instead of money, based on previous studies using similar tasks with children [13, 15, 20].

#### Experimental manipulation

**Fig 1:**
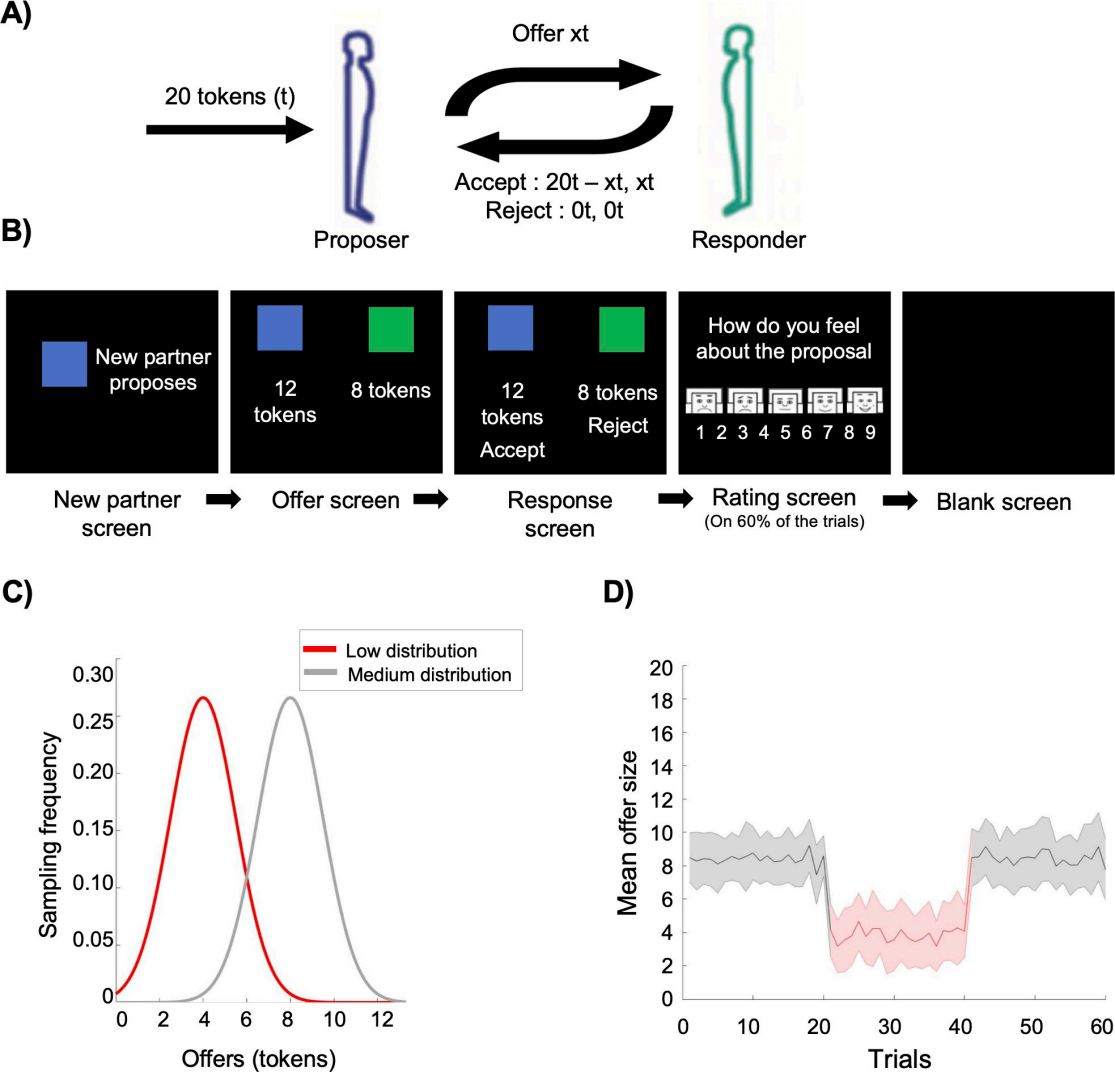
Visual representation of the Ultimatum game. (A) Representation of the exchange process between the Proposer and the Responder. (B) Visual display of the task. Each trial starts with the presentation of a new partner followed by the offer presentation after which children were asked to choose between accepting and rejecting the offer. For 60% of the trials, children had to rate their feeling regarding the offer received using the visual scale shown in the Rating screen. (C) Offers were sampled from two Gaussian distributions. Baseline and Post-conditioning offers were sampled from the medium distribution (mean 8 tokens, SD 1.5 tokens) and conditioning offers were sampled from de low distribution (mean 4 tokens, SD 1.5 tokens). (D) Average offers received throughout the task.

Although several studies with children often used a third-party punishment approach [40] to limit the retaliatory behaviors, Leibbrandt and López-Pérez [41] have found that, compared to second parties, third parties do not punish more normatively and impartially. Nonetheless, in the current study different anonymous proposers were used to reduce the possibility of retaliation actions (i.e., refuse the next offers to punish the proposer). Given the hypotheses of the current study, it was deemed preferable to use a second-party approach as it provides behavioral measures for each child (i.e., rejection rate) and allows for behavioral comparison. At the beginning, children were informed that they would play the role of the Responder and receive offers they had to accept or reject from 60 different children (Proposers) who had to split 20 token endowments (see Fig 1A and 1B). Further, to ensure that each exchange would be treated equally, children were informed that their final payment would be based on the amount of tokens from a randomly selected offer and that the tokens could be used to “buy” a small gift (e.g., pencil, ball) at the end of the experiment. Unknown to them, the offers were controlled by a computer algorithm. The offers were generated from two offer distributions (see Fig 1C and 1D): (1) medium /“fair” offers (*M* = 8 tokens, *SD* = 1.5 tokens) and (2) low /“unfair” offers (*M* = 4 tokens, *SD* = 1.5 tokens). For trials 1 to 20 (Baseline block), children received offers from the medium distribution. For trials 21 to 40 (Conditioning block), they received offers from the low distribution. Finally, for trials 41 to 60 (Post-conditioning block), they again received offers from the medium distribution. Following 67% of the offers, children were asked to rate their emotional reaction regarding the current offer from unhappy to happy on a 9-point Likert scale. To help children understand the scale, emoticons adapted from the self-assessment manikin were used to represent the continuum from unhappy to happy [42]. Previous studies using a similar paradigm suggest that showing several offers from different proposers can induce a conditioning effect on participants’ norms operationalized by differences in rejection rates [10, 11, 32]. Based on previous studies [10, 11, 31, 32] it was posited that if children are able to adapt their expectations (i.e., change their norm) about the kind of offers they should receive, seeing several low offers should make them less likely to reject medium “fair” offers and thus their average rejection rate should be lower in the Post-conditioning block compared to the Baseline block.

### Procedure

The UG was part of a neuropsychological battery lasting 3 hours (other tasks not reported here, as the present study is part of a longitudinal study). The UG was administered after a 15-minutes break (approximately 1.5 hours into the evaluation session) and lasted about 20 minutes. The UG task was presented as an online game played simultaneously with other children of the same age. The following instructions were presented verbally by the examiner:

“*In this activity*, *you will play against several other children your own age*. *The children are also in a room with examiners*, *just like us*. *These children have all been given 20 tokens that they must divide with you*. *They have to decide how many tokens they keep and how many they give you*. *For each offer that you will receive*, *you will have the choice to accept or reject the offer*. *If you accept the offer*, *you and the other child will both get your part of the offer*, *however*, *if you reject the offer*, *you will both receive 0 token*. *To accept the offer*, *you have to press 1 on the keyboard and to refuse the offer you have to press 2*. *If you look at the screen here*, *you will see that there are 2 squares*, *one green and one blue*. *The number under the green square will always represent the number of tokens that the child has decided to keep for himself and the number under the blue square will always represent the number of tokens he is offering you*. *You will receive different offers*, *each offer is made by a different child*. *After some offers*, *you will have to indicate how you feel about the offer you just received with the scale you see on the screen right now*. *You have to choose how happy you are about the offer from 1-unhappy to 9-happy*. *Finally*, *at the end of the game*, *I will randomly draw an offer from all the ones you have received*, *and I will give you the number of tokens so that you can exchange it for a gift in my surprise box*.*”*

Before starting the task, children were asked to explain in their own words what they had to do during the task and how their tokens payment would be determined. Instructions could be repeated as often as necessary until the child fully understood the task and the payment method. Children were also presented with three examples of possible splits (6–14; 8–12; 9–11) and were asked to place them in ascending order, from the one with the smallest discrepancy to the one with the largest (9–11; 8–12; 6–14). This ensured that children were able to compute and order the magnitude of differences between numbers that would be used in the task. All children answered the questions correctly. Two examples were then presented to the child on the computer to ensure that the child understood the task. In these examples, the child was asked to indicate what number represents his or her number of tokens, which keys to press to accept or reject the offer, and how to use the emotion scale. To maintain the child’s attention throughout the task, the examiner sat next to the child and intervened as needed (e.g., “stay focused on the game”, “keep going”).

### Behavioral data analysis

Norm adaptation was assessed for each participant by comparing their rejection rate in the Baseline block and in the Post-conditioning block, given that both blocks presented offers taken from the same medium offers distribution. The same approach was used to assess the potential differences in emotional reactions between the Baseline and Post-conditioning blocks.

### Statistical analyses

Statistical analyses were performed using SPSS 26.0 software and computational analyses were performed using a custom MATLAB R2019a (v9.6.0) script. Prior to all statistical analyses, data were examined for violations of test assumptions (normality, linearity, homogeneity and independence) and all assumptions were respected. No difference between boys and girls or correlations with age were found on any of the study variables (see Table 1) and therefore main analyses were conducted without controlling for participants’ age or sex. First, as a preliminary and exploratory step, a mixed effects logistic regression was conducted to investigate the degree to which the offer size predicted the participant’s decision to accept or reject the offer. In this model, fixed effects represented offer sizes, whereas random effects reflected subject-specific random slopes for the first offer and the subsequent offers. For the primary analyses, paired sample t-tests comparing average rejection rates between Baseline and Post-conditioning blocks across (a) and between (b) offer sizes were performed to investigate children’s ability to adapt their fairness norm to a changing environment. Paired sample t-tests were also used to compared emotional reaction levels between Baseline and Post-conditioning blocks. The strength of effect sizes were determined according to Cohen’s criteria [43].

**Table 1:** Preliminary analyses with model-free and model-based variables, age and sex.

Variable	*M*	*SD*	Correlation with age (*r*)	Sex difference (*t*)
Behavioural adaptation	1.55	2.15	-.14	0.26
Emotional adaptation	2.21	1.89	-.02	-0.22
Envy	2.58	2.53	.16	1.9
Guilt	0.17	0.32	-.17	0.45
Temperature	1.78	1.58	-.19	0.43
Learning rate	0.16	0.28	-.29	0.14

Note. * *p* < .05

Based on the adaptation pattern observed in the UG task (see Results), it was posited that participants’ have an internal representation of the fairness norm that can be modified in a changing social environment [9–11, 31]. Based on previous studies it was assumed that participants’ choices are driven by their aversion to unfair offers and that the utility (i.e., subjective value) of each offer could therefore be modeled by the Fehr-Schmidt inequality aversion model (FS model, 1999).

$$U_{t}\left(s_{t}\right)=s_{t}-\alpha \;\max\;\left\{norm-s_{t},0\right\}-\beta \;\text{max}\;\left\{s_{t}-norm,0\right\}.$$

Here, *U*_*t*_ represents the utility of offer *s*_*t*_ at round *t*. The value of *U* is dependent on the discrepancy between the offer (*s*_*t*_) received by the participant and his internal norm (expected offer)—the norm-prediction error [9]. The disutility is controlled by two free parameters: *α* or “envy” (*α* ∈ [0, 10]), that is, the participant’s unwillingness to accept unequal offers that are disadvantageous to them (i.e., *s*_*t*_ < norm) and *β* or “guilt” (*β* ∈ [0, 10]), that is, the participant’s unwillingness to accept unequal offers that are advantageous to them (i.e., *s*_*t*_ *>* norm). It is important to note that the parameters "envy" and "guilt" do not represent feelings towards the Proposer, but rather the participants’ sensitivity (i.e., unwillingness to accept) to offers that are less than their expectation (envy) or that exceed their expectations (guilt).

The probability of accepting each offer is given by:

$$P_{t}\left(s_{t}\right)=\frac{e^{U*\gamma }}{1+e^{U*\gamma }}$$

Where *γ* (*γ* ∈ [0,1]) represents the softmax inverse temperature parameter that controls the degree of stochasticity in the decision. The lower the value of *γ*, the more diffuse and variable the choices are (i.e., the less the participant will base their choices on the utility function).

In this study, the behavioral data were fitted with two types of norm adaptation models–Bayesian observer models and Rescorla-Wagner models–used in similar studies with adults [10, 11], in addition to a FS model without any norm adaptation (non-learning model). These models were selected based on prior work that used model-based analysis with this version of the UG in adults [10].

#### Bayesian observer model

The Bayesian observer model (BO model) [10] assumes that in the UG task, participants are Bayesian observers who expect each offer to be sampled from a Gaussian distribution with an uncertain mean and variance and that they operate a Bayesian update after each new offer they receive throughout the task. More precisely, it is assumed that each participant has a prior on the distribution of the offers (*s*) where the mean and variance are described as follows: *s~N*(*μ*, *σ*^2^). The mean and the variance are mixed together, therefore the prior of offers (*s*) is assumed as *ρ*(*μ*, *σ*^2^). After receiving an offer *s*_*t*_ at trial *t*, participants update their expectation about *μ*, *σ*^2^ following Bayes’ rule. The posterior is given by:

$$\rho \left(\mu ,\sigma^{2}|s_{t}\right)=\frac{\rho \left(s_{t}|\mu ,\sigma^{2}\right)\rho \left(\mu ,\sigma^{2}\right)}{\rho \left(s_{t}\right)}$$

A conjugate prior is assumed for *μ* and *σ*^2^:

$$\rho \left(\mu ,\sigma^{2}\right)=\rho \left(\mu ,|\sigma^{2}\right)\rho \left(\sigma^{2}\right),$$

$$\rho \left(\mu |\sigma^{2}\right)=\text{Normal}\left(\hat{\mu ,}{\hat{\sigma }}^{2}/k\right)$$

$$\rho \left(\sigma^{2}\right)=\text{Inv}-\chi^{2}\left(\upsilon ,{\hat{\sigma }}^{2}\right).$$

The initial value of the hyperparameters $k,\;\upsilon \;\text{and}\;{\hat{\sigma }}^{2}$ were set as:

$$k_{0}=4,\upsilon_{0}=10,{\hat{\sigma }}_{0}^{2}=4$$

Values were updated as followed after receiving *s*_*t*_ at trial *t*:

$$k_{t}=k_{t-1}+1,\nu_{t}=\nu_{t-1}+1,$$

$${\hat{\mu }}_{t}={\hat{\mu }}_{t-1}+\frac{1}{k_{t}}\left(s_{t}-{\hat{\mu }}_{t-1}\right),$$

$$\nu_{t}{\hat{\sigma }}^{2}{}_{t}=\nu_{t-1}{\hat{\sigma }}^{2}{}_{t-1}+\frac{k_{t-1}}{k_{t}}{\left(s_{t}-{\hat{\mu }}_{t-1}\right)}^{2}.$$

Importantly, at round *t*, the prevailing norm is approximated by taking the mean of the normal distribution describing the participant’s prior (i.e., *μ*_*t*-1_). Using this measure, the utility of the offer is determined by

$$U\left(s_{t}\right)=s_{t}-\alpha \;\max\;\left\{{\hat{\mu }}_{t-1}-s_{t},0\right\}-\beta \;\text{max}\;\left\{s_{t}-\;{\hat{\mu }}_{t-1},0\right\}.$$

In this study, two variations of the BO model were tested. For the first variation, it was assumed that the initial fairness norm for all participants represented equality and therefore ${\hat{\mu }}_{0}$ was fixed at 10. The second supposed that participants could all have a different initial fairness norm, thus, ${\hat{\mu }}_{0}$ was a free parameter that was fitted for each participant using their responses (${\hat{\mu }}_{0}\in \left[0,20\right])$.

#### Rescorla-Wagner model

The Rescorla-Wagner model (RW model) [44, 45] is based on the premise that participants have an internal representation of the norm that can be updated by the RW rule as follow:

$$x_{t}=x_{t-1}+\varepsilon \left(s_{t}-x_{t-1}\right)$$

Here, the norm at trial *t* is represented by *x*_*t*_ and the norm learning rate (*ε* ∈ [0,1]) is represented by *ε*. The norm learning rate–specific characteristic of the RW model–is a free parameter optimized for each participant which represents the extent to which the participant’s norm is influenced by the discrepancy (i.e., norm-prediction error) between the current offer *s*_*t*_ and what was expected based on their preceding norm *x*_*t*_−1. A high *ε* means that the norm-prediction error had a large impact on the norm updating, whereas a low *ε* indicates a smaller impact.

The utility of the offer is determined by

$$U\left(s_{t}\right)=s_{t}-\alpha \max\;\left\{x_{t-1}-s_{t},0\right\}-\beta \;\text{max}\left\{s_{t}-x_{t-1},0\right\}.$$

As with the BO models, two variations of the RW model were tested, the first, where the initial fairness norm *x*_0_ represents equality (*x*_0_ = 10) and the second, where the initial norm can vary across participants (${\hat{\mu }}_{0}\in \left[0,20\right]$).

#### Modified Fehr-Schmidt model

Similar to previous studies on social norm learning [10, 11, 31], a modified version of Fehr-Schmidt inequity aversion model combining the original model and the norm-based utility function proposed by Bichhieri was used [3, 46]. In this model the utility function of each offer at round *t* was represented by the discrepancy between the norm (i.e., what is expected) and the offer received. The utility of each offer is given by:

$$U_{t}\left(s_{t}\right)=s_{t}-\alpha \max\;\left\{norm-s_{t},0\right\}-\beta \;\text{max}\left\{s_{t}-norm,0\right\}.$$

Since the Fehr-Schmidt model is originally a non-learning model, a fixed norm model where the norm does not change (always 10 tokens) was first tested. To match the BO and RW models, a variation of the FS where the initial fairness norm can vary across participants was also tested (*norm* ∈ [0,20]).

Both variations of the BO, RW and FS models were fitted to the behavioral data and the values of envy (*α*), guilt (*β*) and temperature (*τ*) (in addition to learning rate (*ε*) and initial norm (${\hat{\mu }}_{0}$) for certain models) were estimated individually for each participant by maximizing the log likelihood of choice over 60 trials. This procedure was completed for each model. To determine which model better fit the data, they were compared by calculating the Bayesian information criterion score (BIC) and the Akaike information criterion (AIC) across all participants for each model. The winning model was the one with the lowest BIC and AIC sums.

## Results

### Relation between choice and offer size

**Fig 2:**
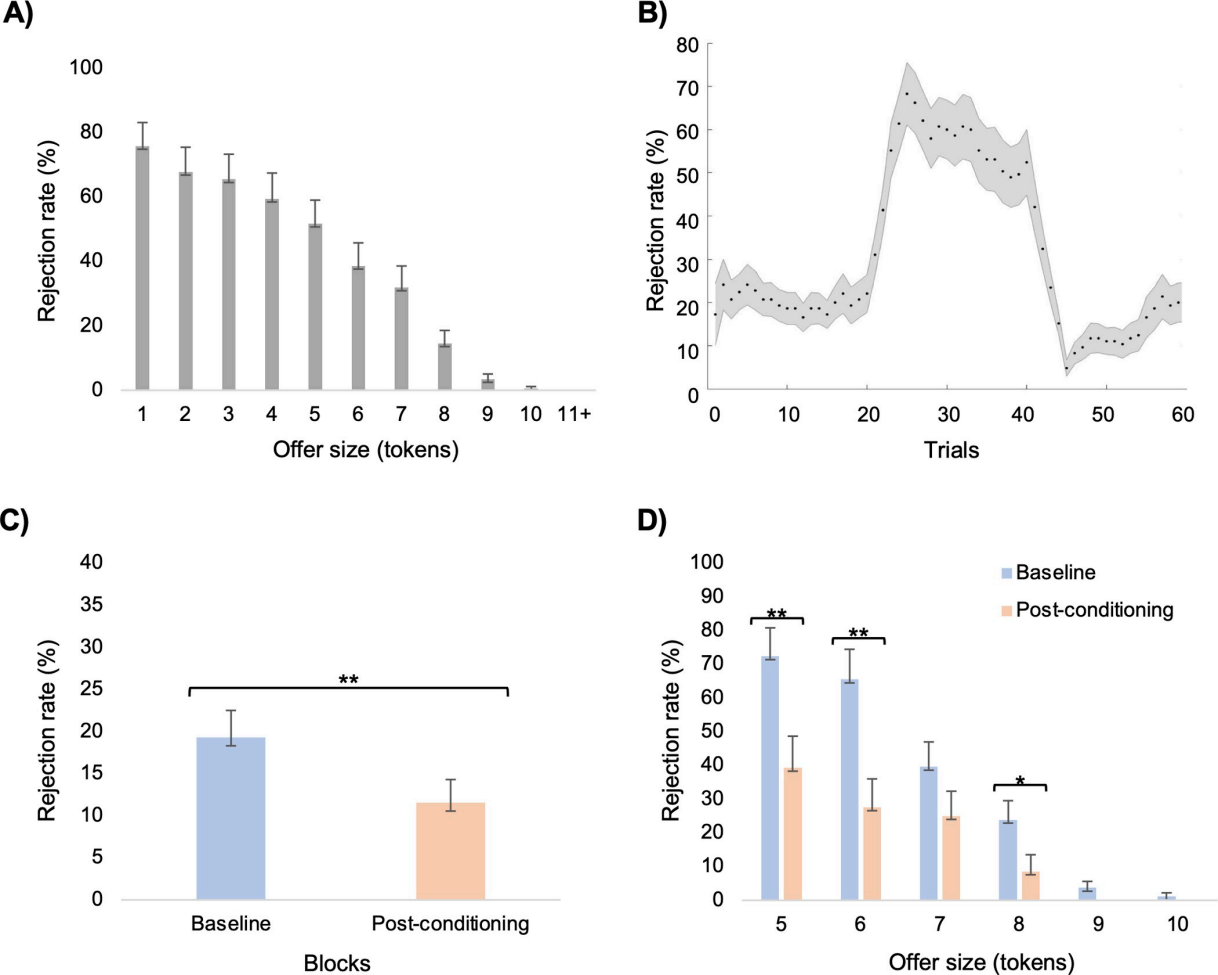
Behavioural results. (A) Percentage of rejection rates for each offer size across the 60 trials. (B) Visual representation of dynamics of the mean rejection rates (%) throughout the task. Rejection rates were calculated with a sliding window of 5 trials. (C) Comparison of mean rejection rates between Baseline and Post-conditioning blocks (D) Comparisons of Baseline and Post-conditioning rejections rates grouped by offer size. Data are represented as mean ± SEM *p < 0.05 **p < 0.01 corrected for multiple comparison when appropriate.

As shown in Fig 2A, there is a negative relationship between mean rejection rates and offer sizes across the 60 trials. A mixed effects logistic regression model was used to investigate this relationship across participants’ data. Results showed that the offer size significantly influences the probability of rejecting the offer within this group (*β* = 1.10; 95% CI[0.73, 1.47], *z =* 5.86, *p* < .001, 95% CI[0.73, 1.47), with an odd ratio of 3.00 (*p <* .*001*, CI 95% [2.07, 4.33]). This suggests that children did not use a “cut-off” strategy by which they would reject everything that is lower than 10 tokens, but rather a more sophisticated utility function at least partly based on the offer size, akin to what has been observed in adults [10, 47].

### Behavioral adaptation

As expected, a significant difference was found between rejection rates Pre and Post conditioning (*t*(29) = -3.89, *p* < .01) with a moderate effect size (*d* = 0.71; 95% CI [0.31, 1.12]). On average, participants’ rejection rates decreased by 7.75% (*SEM =* 1.99) in the Post-conditioning block compared to the Baseline block (see Fig 2C). However, no difference was found between emotional reactions Pre and Post conditioning (*t*(29) = 0.63, *p* = .54). On average children rated their emotional reactions at 7.12 (*SEM* = 0.27) in the Post-conditioning block and 7.30 (*SEM* = 0.26) at Baseline.

Given the significant difference between rejection rates Pre and Post conditioning, further paired-sample t-tests were used to investigate for which offer size differences in rejection rates could be observed between those two blocks. The Benjamini-Hochberg procedure [48] was applied to control for multiple hypotheses testing in order to limit the Type I error (see Table 2; FDR = .05). As shown in Fig 2D, after correction, significant differences were found for the offers of 5 tokens (*t*(29) = -3.58, *p* < .01; *d* = 0.68; 95% CI [0.26, 1.08]), 6 tokens (*t*(29) = -4.14, *p* < .01; *d* = 0.77; 95% CI [0.35, 1,18]) and 8 tokens (*t*(29) = -2.44, *p* = .02; *d* = 0.45; 95% CI [0.07, 0.83]), but not for the offers of 7 tokens (*t*(29) = -2.14, *p* = .04) and 9 tokens (*t*(29) = -1.95, *p* = .06). This means that for the offer 5, 6 and 8 tokens, children generally rejected less offers in the Post-conditioning block than in the Baseline block.

**Table 2:** Benjamini-Hochberg correction.

Rank	Offers	P-value	(i/m)Q
1	5	.001	.01
2	6	.001	.02
3	8	.021	.03
4	7	.041	.04
5	9	.062	.05

*Note*. The Benjamini-Hochberg Procedure works as follows: 1) The individual p-value for each test are put in ascending order; 2) Rank is assigned to each p-value from smallest (rank 1) to largest (here rank 5); 3) The Benjamini-Hochberg critical value is calculated for each p-value using this formula: (rank of p-value/total number of tests)*the chosen false discovery rate; 4) The largest original p-value that is smaller than the Benjamini-Hochberg critical value is identify and represents the cut-off point; 5) Every p-value smaller than the cut-off point is considered to be significant.”

As shown in Fig 2B, in addition to changes between blocks, changes in rejection rates seems to occur within each of the three blocks. This observation is consistent with the hypothesis that children have an internal model of their norm that they update according to the new information they receive. Moreover, fluctuations in rejection rates within the same block—especially salient in the conditioning block–suggest that the internal norm is continuously updated, even when the information received are similar (here, from the same distribution of offers). These observations suggest a dynamic learning process for which we next sought to uncover the computational mechanism.

### Behavioral model

**Fig 3:**
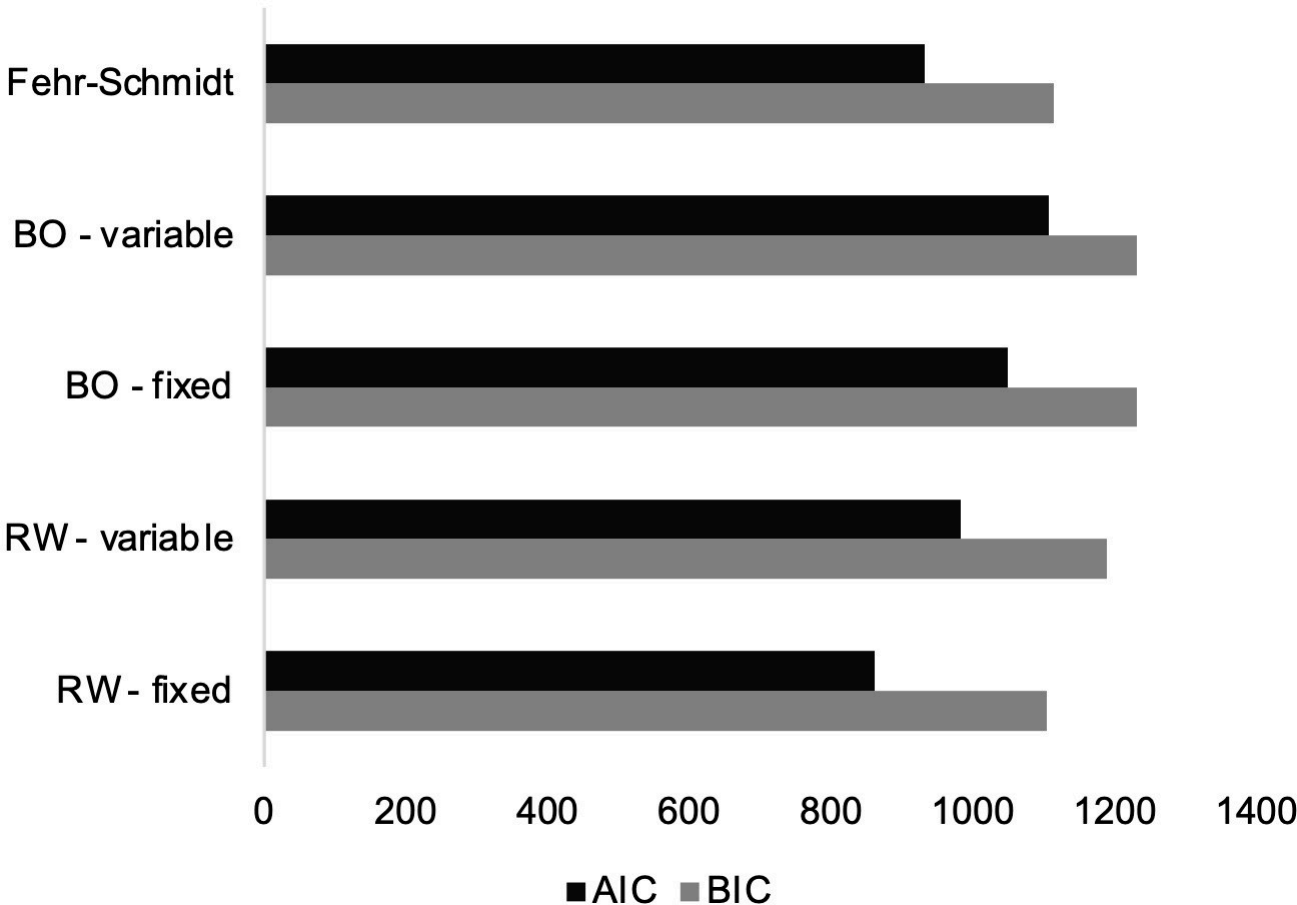
Sum BIC and AIC scores for behavioral models.

**Fig 4:**
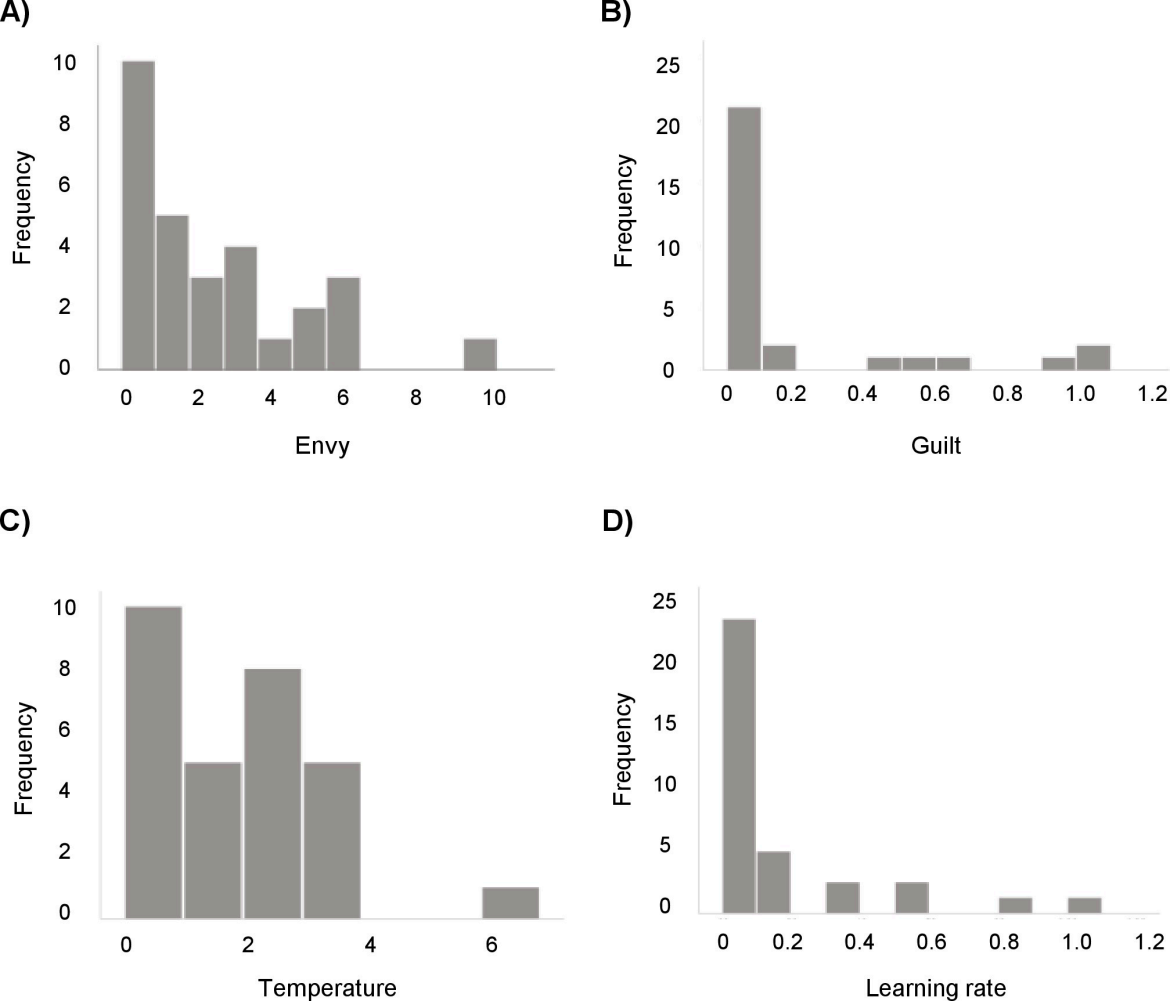
Distribution of the free parameters for the winning model.

The Rescorla-Wagner with a fixed initial norm (*x*_0_ = 10) was identified as the winning model as it had the lowest BIC as well as the lowest AIC (see Fig 3). Table 1 presents the winning model’s parameter estimates statistics, whereas Fig 4 displays their distribution.

## Discussion

The primary aim of this study was to investigate children’s abilities to adapt their fairness norm based on implicit social information in a changing social environment using a conditioning paradigm. A second objective was to identify the computational mechanism controlling this adaptation process. The findings indicate children rejection rates for medium offers decreased after being exposed to several low offers, which is consistent with the hypothesis that children aged between 7 and 11 years old can sample and use implicit social information in order to adapt their fairness norm. The model-based analysis revealed that, in this specific sample, this dynamic process seems to be governed by a mechanism by which children compute the difference between their expectations and the offer they receive–a norm-prediction error signal–to update their fairness norm. Together, these findings significantly add to the body of knowledge regarding the internal process underlying norm adaptation abilities, specifically regarding the fairness norm, in children.

Given that the present study was one of the first to use this experimental version of the UG in a pediatric population, we first focused on the children’s overall behaviors across the entire task. Results suggest that offer size plays a predictive role in children’s decision-making process in the UG (i.e., accept vs. reject the offer received) with decreasing offer sizes associated with increased probability of rejection. On closer examination, as seen in Fig 2A, rejection rates seem to increase as a function of how much the offer differs from an equal division (i.e., offer < 10 tokens). This pattern of rejection is similar to what is generally observed in adults (see behavioral results from Gabay et al., 2014’s meta-analysis on the UG) and is consistent with the inequality aversion model [46], which posits that most individuals, including children, have a preference for equal distribution of resources that leads them to punish fairness transgression, even when it implies a personal cost for themselves [19]. Therefore, the present findings are consistent with previous studies and suggest that by mid-childhood, children do tend to enforce the fairness norm by punishing transgressors [13, 14, 20]. Since most previous studies have only compared children’s decisions between very unfair and fair offers [13, 14, 19], by presenting a wide range of unequal offers we were also able to show that children’s decision-making is not simply based on a dichotomous analysis of fairness compliance (i.e., is it fair or not?) that would result in rejecting all offers that diverge from fairness. Instead, similarly to what is observed in adults [10, 11, 31], children’s decision-making appears to be underpinned by a complex process that considers the extent of the fairness violation [9]. The methodology used in the present study thus helps provide a deeper understanding of fairness norm processing in children.

In line with the study’s main hypothesis, children rejected significantly fewer medium offers after being exposed to an environment that presented multiple low offers. The strength of this adaptation effect showed no association with sex or age. Further analyses indicate that, after correcting for multiple comparisons, this decrease in rejection rate remained significant for almost all offer sizes representing an unequal split. Thus, this study is the first to establish that by 7 years, children seem to use implicit information present in their environment to update their expectations (fairness norm) and adjust their behavior accordingly. These changes in rejection rates for otherwise identical offers are consistent with a norm conditioning effect found in adults [10, 11] and strongly suggest a change in children’s internal expectations (i.e., social norm) [3]. To date, most of the studies investigating children’s norm or behavioral adaptation capacities have done so in relation to explicit demands or social pressure to conform [23, 27, 28, 49]. Conversely, the present study emphasizes that children are able to do this in the absence of explicit information or instructions (e.g., opinion, suggestion or decision of a third party). Interestingly, although children reject lower offers less frequently, they do not appear to be more satisfied with them as reflected by the lack of change in subjective ratings. While this result could be a true absence of emotional impact, it is also possible that methodological limitations hampered our ability to detect negative emotions towards lower offers, as seen in adults [10, 11]. Indeed, self-report measures of subjective feeling about offers does not optimally capture affective changes in children [50]. In sum, using a conditioning paradigm, this study suggests that being exposed to a changing social environment can lead to changes in children’s cognitive representation of the fairness norms which can be observed by changes in their behaviors during social interactions characterized by resources allocation.

The second objective of this study was to identify the computational mechanism underlying norm adaptation. Our data showed inter-and-intra block changes in rejection rates, suggesting that norm adaptation was controlled by a dynamic learning process that updates the fairness norm based on the offers received, as described in adults [10, 11, 31]. Comparisons of different learning and non-learning models showed that the dynamic process was not better explained by a non-learning model with a fixed value representing the fairness norm (e.g., 10 tokens). Instead, in this sample, the decrease in rejection rates for identical offers (i.e., medium offers Pre and Post conditioning), as well as the trial-by-trial change in rejection rates within blocks observed in this study, are most likely explained by a Rescorla-Wagner-based model [44] with a fixed initial fairness norm: a learning model where the internal representation of the fairness norm is continuously modified throughout the task [9–11, 31]. This model proposes that children initially expect to receive offers that represent an equal split between them and the other player, but that their representation of the fairness norm is then updated using a RW rule. Specifically, on a trial-by-trial basis, receiving offers that do not ’match’ their expectations lead to computation of a norm-prediction error that is subsequently used to update the norm [9]. This update is controlled by a learning rate parameter that can vary between individuals, where the greater the learning rate the more influence the norm-prediction error has on the norm or, in other words, the faster one adapts. In line with the model-free results, there was no relation between sex or age and learning rate. In the present study, the LR parameter is unique to RW models, which could potentially explain why the RW with a fixed initial fairness norm is the winning model. Indeed, compared to our BO models, which assume that norm update occurs in the exact same way for each participant, RW models incorporate an additional parameter that lets the learning “speed” (or how much importance one puts on new vs. old information) vary between participants. Thus, the fact that the winning model included this parameter could suggest that there are individual differences in how children use new information to update their social norm which could not be captured by our other learning models. Similar norm prediction-based computational mechanisms have been shown to underpin the behavior of adults in comparable UG paradigms [10, 11, 31]. In sum, children’s brains appear to have the ability to compute norm-prediction errors and use this signal to dynamically update their fairness norm in order to adapt to a changing environment.

### Limitations and future directions

Certain limitations need to be addressed in order to properly interpret the results. First, it is important to consider that the small sample size as well as the selected age range prevent the generalization of results to all school-aged children. Further, the current sample only included typically developing children, mostly from upper-middle class families, whose social learning and functioning might differ from children living in more disadvantageous conditions or children with medical or psychological diagnostics. Hence, it is possible that different results could be obtained in a more diverse sample in terms of age and sociodemographic characteristics. In addition, given the methodology used, the results can only be interpreted with respect to the fairness norm and therefore cannot be generalized to other types of social norms. Further, although results are highly consistent with the hypothesis of norm adaptation, they do not allow for a causal interpretation where changes in behaviors would undeniably means fairness adaptation. Moreover, children were only presented with disadvantageous offers during the conditioning block which did not allow for conclusions regarding their norm adaptation abilities when exposed to unfair but advantageous offers. Given that previous studies indicate that children’s behaviors toward advantageous and disadvantageous offers differ before the age of 8 years [14, 28], it is crucial that future studies use both conditions. Finally, future studies could also use a larger and more diverse sample in terms of age and culture to generalize the results beyond mid-childhood and children from westernized (mainly white and relatively high SES) populations.

## Conclusion

In conclusion, the present study is the first to use an innovative conditioning paradigm and computational modeling to study school-age children’s fairness norm processing and adaptation and their underlying learning mechanisms. Children 7 to 11 years old are able to sample and use implicit social information, detect fairness violations in order to change their behaviors, which suggest the ability to adapt their fairness norm to better fit their environment. Further, this dynamic process seems to be governed by a norm-prediction based learning model. Overall, our model-free and model-based findings provide a deeper understanding of social norm processing during childhood. Future studies could seek to combine this methodology with neuroimaging in order to investigate which neural substrates underpin this norm adaptation mechanism in children, further expanding our understanding of non-optimal social behaviors associated to certain neurological or behavioral conditions.

## Supporting information

S1 Table Descriptive statistics of free parameters. (DOCX)
