# Supplementary material for: How do children adapt their fairness norm? Evidence from computational modeling
Source: PLoS One. 2022 Nov 16;17(11):e0277508. doi: 10.1371/journal.pone.0277508 (PMC9668110; doi:10.1371/journal.pone.0277508)
Supplement: S1 Table — (DOCX) [file pone.0277508.s001.docx]

**S1 Table. Descriptive statistics of free parameters**

|  | Envy | | Guilt | | Temperature | | Learning rate | | Initial norm | |
| --- | --- | --- | --- | --- | --- | --- | --- | --- | --- | --- |
| Model | *M* (*SD*) | Range | *M* (*SD*) | Range | *M* (*SD*) | Range | *M* (*SD*) | Range | *M* (*SD*) | Range |
| FS - variable | 3.38 (3.65) | 0 - 10 | 0.44 (0.34) | 0 - 1 | 1.51 (1.39) | 0 - 5.59 | - | - | 10.07 (5.45) | 0 - 20 |
| FS - fixed | 1.52 (1.56) | 0 - 6.45 | 0.26 (0.41) | 0 - 1 | 1.88 (1.56) | 0 - 5.59 | - | - | - | - |
| BO - variable | 3.45 (3.56) | 0 - 10 | 0.08 (0.25) | 0 - 0.99 | 3.06 (2.27) | 0 - 9.20 | - | - | 10.36 (7.17) | 0 - 20 |
| BO - fixed | 3.44 (3.56) | 0 - 10 | 0.10 (0.26) | 0 - 0.98 | 3.06 (2.27) | 0 - 9.20 | - | - | - | - |
| RW - variable | 3.10 (3.45) | 0 - 10 | 0.31 (0.42) | 0 - 1 | 1.41 (1.31) | 0 - 3.97 | .16 (.28) | 0 - 1 | 11.93 (5.38) | 0 - 20 |
| RW - fixed | 2.67 (2.53) | 0 - 10 | 0.17 (0.32) | 0 - 1 | 1.78 (1.58) | 0 - 6.18 | .16 (.28) | 0 - 1 | - | - |
